# Increased Vascular Permeability Due to Spread and Invasion of *Vibrio vulnificus* in the Wound Infection Exacerbates Potentially Fatal Necrotizing Disease

**DOI:** 10.3389/fmicb.2022.849600

**Published:** 2022-03-08

**Authors:** Kohei Yamazaki, Takashige Kashimoto, Takehiro Kado, Kazuki Yoshioka, Shunji Ueno

**Affiliations:** ^1^Laboratory of Veterinary Public Health, School of Veterinary Medicine, Kitasato University, Towada, Japan; ^2^Department of Microbiology, University of Massachusetts, Amherst, MA, United States; ^3^Laboratory of Veterinary Anatomy, School of Veterinary Medicine, Kitasato University, Towada, Japan

**Keywords:** *Vibrio vulnificus*, necrotizing soft tissue infection (NSTI), pathogenic factor, vascular permeability, murine model, *in vivo* imaging

## Abstract

*Vibrio vulnificus* is known to cause necrotizing soft tissue infections (NSTIs). However, the pathogenic mechanism causing cellulitis, necrotizing fasciitis, muscle necrosis, and rapidly developing septicemia in humans have not been fully elucidated. Here, we report a multilayer analysis of tissue damage after subcutaneous bacterial inoculation as a murine model of *V. vulnificus* NSTIs. Our histopathological examination showed the progression of cellulitis, necrotizing fasciitis, and muscle necrosis worsening as the infection penetrated deeper into the muscle tissue layers. The increase in vascular permeability was the primary cause of the swelling and congestion, which are acute signs of inflammation in soft tissue and characteristic of human NSTIs. Most importantly, our sequential analysis revealed for the first time that *V. vulnificus* not only spreads along the skin and subcutaneous tissues or fascia but also invades deeper muscle tissues beyond the fascia as the crucial process of its lethality. Also, increased vascular permeability enabled *V. vulnificus* to proliferate in muscle tissue and enter the systemic circulation, escalating the bacterium’s lethality. Our finding may yield important clinical benefits to patients by helping physicians understand the impact of surgical debridement on the patient’s quality of life. Furthermore, this study provides a promising system to accelerate studies of virulence factors and eventually help establish new therapies.

## Introduction

*Vibrio vulnificus* is a Gram-negative and highly motile bacterium that lives in the sea and brackish waters and is known as a major causative bacterium of necrotizing soft-tissue infections (NSTIs; Tacket et al., 1984; Klontz, 1988; Finkelstein et al., 2002; Ito et al., 2012; Oliver, 2015; Baker-Austin and Oliver, 2016; Stevens and Bryant, 2017; Yamazaki et al., 2019). There are two types of *V. vulnificus* infection: primary septicemia and wound infection. Primary septicemia generally develops following ingestion of raw, contaminated seafood in patients with underlying disease and leads to death (Klontz, 1988; Ito et al., 2012; Stevens and Bryant, 2017). Wound infection occurs when an open wound is exposed to *V. vulnificus* in seawater, even in healthy people with no underlying disease (Tacket et al., 1984; Finkelstein et al., 2002; Oliver, 2015; Baker-Austin and Oliver, 2016). Moreover, a greater incidence of wound infections caused by *V. vulnificus* has been reported, owing to rising seawater temperatures caused by climate change (Baker-Austin et al., 2010, 2013; Hakkarainen et al., 2014).

Swelling, edema, cellulitis, and necrotizing fasciitis in soft tissues of limbs known as NSTIs are common signs of *V. vulnificus* infections arising from an open wound exposed to the bacterium in seawater or ingested in contaminated food, leading to primary septicemia (Klontz, 1988; Finkelstein et al., 2002; Ito et al., 2012; Hakkarainen et al., 2014; Oliver, 2015; Baker-Austin and Oliver, 2016; Stevens and Bryant, 2017; Yamazaki et al., 2019). The local signs are regarded as a chain of events that are associated with increased vascular permeability (Hakkarainen et al., 2014; Oliver, 2015), and the edema arising from it predisposes patients to cellulitis and necrotizing fasciitis (NF; Swartz, 2004; Lewis et al., 2005; Sarani et al., 2009; Roje et al., 2011; Stevens and Bryant, 2017). Cellulitis is a severe presentation of aggressive infection of deep soft tissue (Swartz, 2004; Lewis et al., 2005; Roje et al., 2011). The acute inflammatory response begins with neutrophil migration to the subcutaneous tissue and progresses to necrotizing fasciitis when inflammation reaches the fascia (Sarani et al., 2009). The clinical signs in wound infection resulting from the *V. vulnificus* infection are known to develop within 16 h, and over 80% of patients have no underlying disease (Oliver, 2015). Taken together, these suggest that *V. vulnificus* has an efficient mechanism for proliferating in soft tissue and evading the immunity of a healthy host. It does this through multifunctional autoprocessing repeats-in toxin (MARTX) and encapsulation, which helps resist phagocytic cells (Lo et al., 2011; Roje et al., 2011; Jeong and Satchell, 2012). *Vibrio vulnificus* strains isolated from the environment have *rtxA*, which encodes MARTX but exhibits a translucent colony, meaning it is not encapsulated in contrast to clinical strains that are encapsulated (Simpson et al., 1987; Oliver, 2015; Yamazaki et al., 2019; Kashimoto et al., 2021).

To understand the pathogenic mechanism of *V. vulnificus* in patients, *in vivo* models have been developed. Suckling mice and iron-overloaded mice models focused on studying proliferation mechanisms in the systemic circulation (Gulig et al., 2005; Kim et al., 2014; Arezes et al., 2015). However, these models do not focus on proliferation mechanisms in the soft tissues. Hence, we had developed a wound infection model of *V. vulnificus* and used this model to select pathogenic factors and to evaluate pathogenic mechanisms in NSTI (Yamazaki et al., 2019, 2020; Kashimoto et al., 2021). However, there are no reports of detailed analysis of the progressive pathology in wound infection using *in vivo* models. Thus, it is not entirely clear how *V. vulnificus* proliferates in the soft tissue of a healthy host and causes NSTI. Here, we subcutaneously (SC) inoculated healthy mice with the pathogen and quantified the degree of the skin and soft-tissue infection. In our thorough investigation of the murine wound infection model, we successfully observed dose- and time-dependent clinical signs, such as inflammation, edema, cellulitis, and necrotizing fasciitis by a multi-faceted approach. Here, we report for the first time that characteristic clinical signs primarily increased vascular permeability, signaling the spread in soft tissues and invasion in deep tissue by *V. vulnificus*. Our detailed analysis of the infection site may lead to potentially life-saving therapeutics against *V. vulnificus* NSTIs.

## Materials and Methods

### Ethics Statement

All animal studies were carried out in strict accordance with the Guidelines for Animal Experimentation of the Japanese Association for Laboratory Animal Science (JALAS; Science Council of Japan, 2006). The animal experimentation protocol was approved by the president of Kitasato University based on the judgment of the Institutional Animal Care and Use Committee of Kitasato University (Approval No. 17-268).

### Bacteria

*Vibrio vulnificus* CMCP6 and E4 was cultured aerobically in Luria-Bertani (LB) broth or on LB agar at 37°C. When required, the medium was supplemented with rifampicin (50 μg/ml) or ampicillin (100 μg/ml) for *V. vulnificus*.

### Mutant Construction

The gene deletion mutant was constructed as described previously (Yamazaki et al., 2020). Briefly, the franking regions, 1,005 bp of upstream and the 1,040 bp of downstream, of *rtxA* gene were amplified with primers: RTX up Fw (5'-GGA TCC TTG CCT TTT AAT TGA GCT TGC-3'), RTX up Rev (5'-TCT AGA TCT CAT TTA CCC TGT GAA GAA GTA TTC AAC-3'), RTX down Fw (5'-TCT AGA TGC TAA CGC ATG TTG GTG ATG-3'), and RTX down Rev (5'-GCA TGC ATG AGT AAT GAT GTT GGC TTT-3'). The underline shows the recognition sites of the restriction enzymes, *BamH*I, *Xba*I, and *sph*I, respectively. The amplicons were confirmed by Sanger sequencing, then digested with *Bam*HI-*Xba*I and *Xba*I-*Sph*I, and cloned into the suicide vector pYAK1, retaining *sacB*. *E. coli* BWλpir were used as a conjugal donor to *V. vulnificus* CMCP6 with spontaneous rifampicin resistance. pYAK1-*rtxA*KO was introduced into *V. vulnificus* CMCP6. *Vibrio vulnificus* retaining pYAK1-*rtxA*KO was cultured in LB broth containing 20% sucrose following the standard *sacB*-assisted allelic exchange method. Mutants were confirmed by PCR to detect expected changes in size at the *rtxA* locus. The plasmid pXen-13 containing a bacterial luminescent gene cluster (*luxCDABE*) was transformed into *V. vulnificus via* electroporation. *Vibrio vulnificus* was grown in LB medium supplemented with 100 μg/ml ampicillin with agitation (163 rpm) at 37°C.

### Mice

Five-week-old female C57BL/6 and BALB/c mice were purchased from Charles River Laboratories Japan (Atsugi, Japan). C57BL/6 mice and BALB/c mice were bred and maintained under specific pathogen–free conditions at Kitasato University. The mice were housed in plastic cages in a group and were maintained on a standard laboratory diet (rat chow MF, Oriental Yeast Co., Ltd. Tokyo, Japan) and tap water under a 12 h light and dark cycle. The ambient temperature during the study was maintained at about 21°C.

### Vascular Permeability Assay

Thirty minutes prior to euthanasia, infected mice were injected intravenously (IV) with 0.1 ml of 5 mg/ml Evans blue dye (Wako, Osaka, Japan). Whole thighs of the sacrificed mice were transected at the ankle and at the hip joint, peeled, divided into SC tissue with skin and muscle tissue, and incubated at 60°C for 24 h in three volumes of formamide. Quantitative analysis of extracts containing Evans blue dye was determined by measuring optical density of blue at 600 nm (OD_600_) with a microplate reader (Sunrise/TECAN Japan, Kanagawa, Japan; Nakamura et al., 2013; Radu and Chernoff, 2013).

### Histopathological Examination

Infection sites excised from sacrificed mice were demineralized by immersing them in buffer solution containing 0.2 M EDTA-4Na and 1% formalin for 1 week, fixed in 10% buffered formalin for 1 day, embedded in paraffin, sliced into 3-μm sections, and stained with Hematoxylin–eosin (H&E). For immunofluorescence staining, the paraffin-embedded sections were used. After deparaffinization, heat-based antigen retrieval and endogenous peroxidase blockade using 3% hydrogen peroxide were performed. Rabbit anti-neutrophil elastase (1:100, ab68672; Abcam) was added and incubated overnight at 4°C. Following a rinse in PBS, peroxidase (PO)-conjugated goat anti-rabbit IgG (Histofine Simple Stain Mouse MAX-PO, Nichirei Bioscience, Tokyo, Japan) was added and incubated for 1 h at room temperature. The site of the immunoreaction was visualized by the diaminobenzidine (DAB) reaction. Sections were counterstained with Mayer’s haematoxylin and mounted under coverslips. Image acquisition of the muscle tissue was performed using an inverted microscope (DM2500/Leica Microsystems, Tokyo, Japan) equipped with 5×/0.40, 10×/0.40, 20×/0.40, and 40×/0.40 objective lenses.

### Evaluation of Biomarkers Following Intramuscular Damage

*Vibrio vulnificus* was grown in LB medium supplemented with 100 μg/ml of rifampicin with agitation (163 rpm) at 37°C. Overnight cultures (100 μl) were inoculated into 2 ml of fresh LB medium and incubated for 2 h. Bacteria were harvested, washed with PBS (pH 7.2) containing 0.1% gelatine, and resuspended in fresh LB medium. Then, 10^6^ colony-forming units (CFU)/mouse were SC inoculated in mice. Infected mice were sacrificed at 9 h post-infection. Whole-blood samples were collected in a syringe by cardiac puncture at multiple time points after infection and centrifuged at 1,200 *g* for 30 min, and sera were collected. Serum creatine kinase (CK), lactate dehydrogenase (LDH), and aspartate aminotransferase (AST) concentrations were evaluated using Dimension EXL with the LM Integrated Chemistry System (Siemens, Tokyo, Japan).

### *In vivo* Bioluminescent Imaging

*Vibrio vulnificus* pXen-13 was grown in LB medium supplemented with 100 μg/ml ampicillin with agitation (163 rpm) at 37°C. Overnight cultures (100 μl) were inoculated into 2 ml of fresh LB medium supplemented with 100 μg/ml ampicillin and incubated for 2 h. Bacteria were harvested, washed with PBS (pH 7.2) containing 0.1% gelatine, and resuspended in fresh LB medium. Then, 10^6^ CFU/mouse were SC inoculated in BALB/c mice. Luminescence signals emanating from *V. vulnificus* were imaged at defined time points using an *in vivo* imaging system (IVIS) 200 imaging system (Xenogen/PerkinElmer, MA, United States) with a 1 min exposure time. The total photons emitted were acquired using the Living Image software package. Mice were anesthetized in chambers containing 2.0% isoflurane inhalant (Pfizer, Tokyo, Japan).

### Kaplan–Meier Survival Curve and Bacterial Burden

*Vibrio vulnificus* was grown in LB medium supplemented with 100 μg/ml of rifampicin with agitation (163 rpm) at 37°C. Overnight cultures (100 μl) were inoculated into 2 ml of fresh LB medium and incubated for 2 h. Bacteria were harvested, washed with PBS (pH 7.2) containing 0.1% gelatine, and resuspended in fresh LB medium. Then, the bacteria were SC, intramuscularly (IM), or intravenously (IV) inoculated in mice. Infected mice were carefully monitored and sacrificed at endpoints to measure survival time or at defined time points to collect tissues. The collected muscles or spleen were suspended in cold PBS containing 0.1% gelatine, homogenized for 5 s with a lab mixer IKA EUROSTAR digital (IKA Werke, Germany; 1,300 rpm), and centrifuged at 800 rpm for 5 min. The supernatants were plated at 10-fold serial dilutions in duplicate on LB agar containing 50 μg/ml rifampicin and incubated for 12 h at 37°C. *Vibrio vulnificus* colonies were counted, and bacterial burden was determined by calculating the number of CFU/g.

### Statistics

Statistical analysis was performed using GraphPad Prism (GraphPad Software, CA, United States). Statistical differences between the two groups were analyzed using the Mann–Whitney *U* test or Fisher’s exact test. Survival curves were analyzed using the log-rank test. A *p* value less than 0.05 was considered significant, and significance values are indicated as follow: ^*^p < 0.05; ^**^p < 0.01; and ^***^p < 0.001.

## Results

### Dose-Dependent Lethality of a Clinical Isolate Strain of *Vibrio vulnificus*

**Figure 1:**
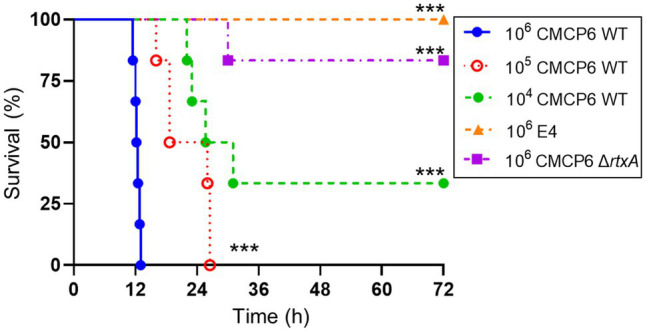
Lethality of *Vibrio vulnificus*. Kaplan–Meier survival curves for mice subcutaneously inoculated with CMCP6 wild type (WT) 10^4^ CFU/head (*n* = 6), WT 10^5^ CFU/head (*n* = 6), WT 10^6^ CFU/head (*n* = 6), E4 10^6^ CFU/head (*n* = 6), and CMCP6 Δ*rtxA* 10^6^ CFU/head (*n* = 6) and monitored for 72 h. ^***^p = 0.0005 compared with CMCP6 WT 10^6^ CFU/head; log-rank test.

*Vibrio vulnificus* NSTI is a fulminant infection that causes death within a few days in humans. The consistent assessment of the process of host death due to the pathogenicity of *V. vulnificus* is a prerequisite for an infection model. First, we investigated whether the wound infection model could assess the lethality of a clinical isolate strain. Clinical strain CMCP6 wild type (WT) and environmental strain E4 were SC inoculated into mice and observed for 72 h. Mice infected with CMCP6 WT caused dose-dependent death (Figure 1). Some mice infected with CMCP6 WT survived in the 10^4^ CFU/head-inoculation group (Figure 1). However, in the 10^5^ CFU/head-inoculation or higher, the survival time was shortened in a dose-dependent manner, and all mice died (Figure 1). On the other hand, mice infected with the environmental strain E4 were asymptomatic and did not die (Figure 1). These results indicate that our murine model can determine the dose-dependent lethality of clinical strains. Besides, some mice infected with 10^6^ CFU/head of CMCP6 Δ*rtxA* survived (Figure 1). Data showed that the murine model has the resistance against *V. vulnificus* proliferation depending on the number of infected bacteria and virulence factors.

### Increased Vascular Permeability at the Bacterial Proliferation Site

**Figure 2:**
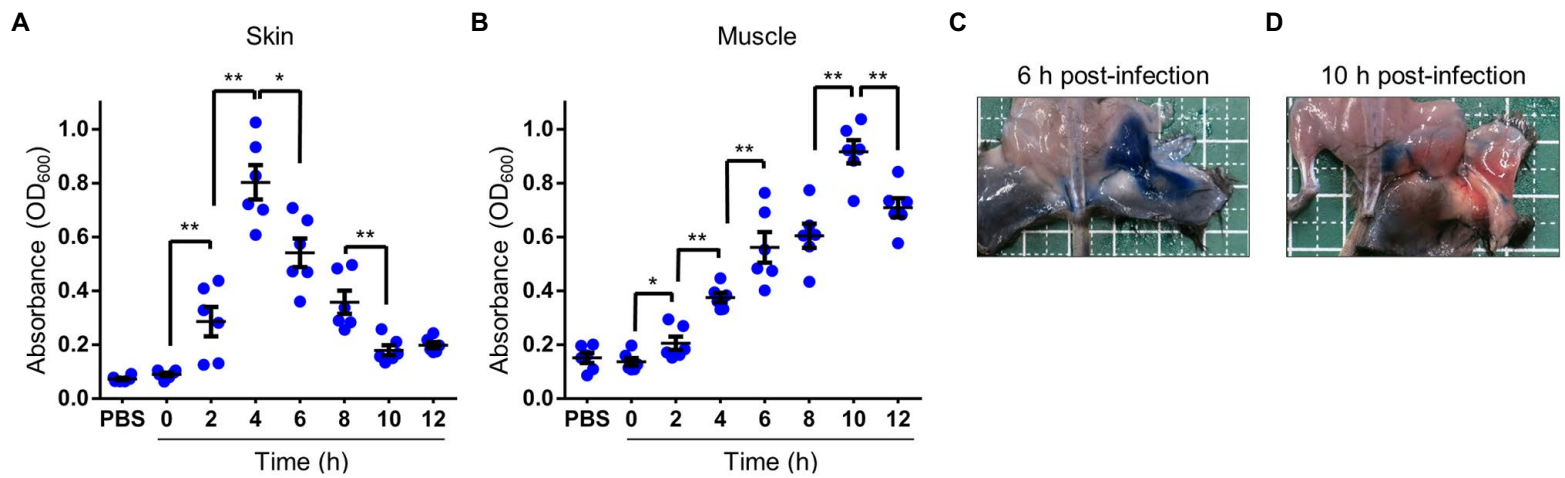
Increased vascular permeability in soft tissues. **(A–D)** Thirty minutes before euthanasia, infected mice were injected i.v. with 0.1 ml of 5 mg/ml Evans blue dye. **(A,B)** Quantification of Evans blue dye extracted from the skin and subcutaneous tissue **(A)** or muscle tissue **(B)** in infected mice. Each symbol represents an individual optical density at 600 nm (OD_600_; *n* = 6/group). Error bars indicate SEM. ^*^p < 0.05; ^**^p < 0.01; Mann–Whitney *U*-test. **(C,D)** Appearance at 30 min after i.v.-inoculation with Evans-blue and 6 h after inoculation **(C)** and 10 h after subcutaneously inoculation **(D)** with wild type (WT). Blue spots caused by extravasation of the Evans blue-serum albumin complex were observed *via* peeling murine skin.

Serum or plasma filling swells and blisters are known signs of *V. vulnificus* infection (Tacket et al., 1984; Klontz, 1988; Finkelstein et al., 2002; Ito et al., 2012; Oliver, 2015), and acute inflammation causes vasodilation and increased vascular permeability. In addition, it has been reported that a protease VvpE of *V. vulnificus* enhances vascular permeability in the guinea pig skin infection model (Jeong et al., 2000; Miyoshi, 2006). We examined the clinical signs and the risk factor during the *V. vulnificus* infection in our experiment, the thighs of mice infected with CMCP6 WT showed severe swelling. Therefore, we investigated whether the swelling was caused by the increased vascular permeability of Evans blue dye that binds to serum albumin immediately following its IV injection into the bloodstream. Under normal physiologic conditions, serum albumin does not cross the vascular endothelial cells, and the Evans blue dye remains restricted within blood vessels. Therefore, only conditions that promote increased vascular permeability allow for extravasation of Evans blue dye at the local tissues. We injected the Evans blue dye into the tail vein of the mice 30 min before harvesting the tissue for observation and measurement of the Evans blue dye extravasation. The time-course analysis by measurement of the leakage of Evans blue dye showed that increased vascular permeability began at 2 h post-infection and peaked at 4 h post-infection in the skin and SC tissue (Figure 2A). In the muscle, it gradually increased and peaked at 10 h post-infection (Figure 2B). Consistently with these results, leakage of Evans blue dye was observed both in the SC tissue and superficial muscle of the local infection site at 6 h post-infection, but not at 10 h post-infection (Figures 2A–D). These results suggested that the Evans blue dye leaked at the inside of deep muscle tissues. Thus, we clearly show that vascular permeability increases in the SC tissue in the early and deeper muscle tissue in the late stages of infection.

### Infiltration of Neutrophils Associated With the Congestion of Blood Vessels

**Figure 3:**
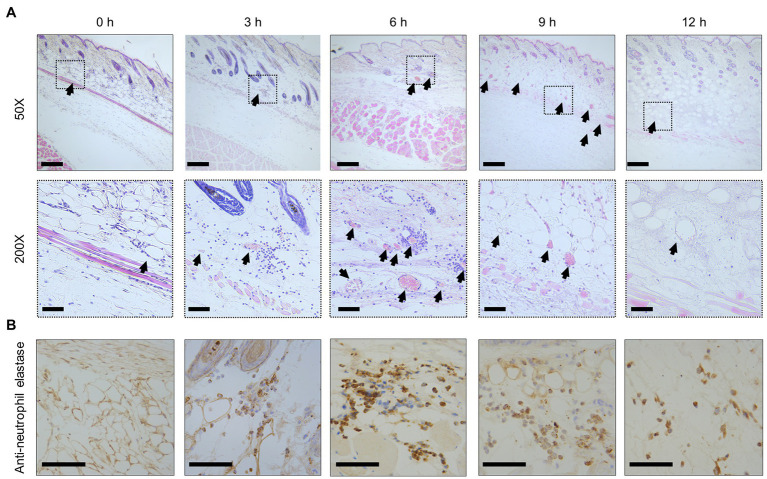
Congestion and inflammation in the skin. **(A)** Hematoxylin–eosin-stained histopathology images at the subcutaneous inoculation site in a mouse infected with *Vibrio vulnificus* (10^6^ CFU/mouse). Lower panels show a high-power field of the part enclosed in upper panels. Arrowheads indicate blood vessels in the subcutaneous tissue. Scale bars: 100 μm (upper panels) and 25 μm (lower panels). **(B)** The immunostained-histopathology images with anti-neutrophil elastase antibody at the subcutaneous inoculation site in a mouse infected with *V. vulnificus* (10^6^ CFU/mouse). Scale bars: 25 μm.

With inflammation, increased vascular permeability promotes the effusion of cellular components and plasma, such as albumin, immunoglobulins, and fibrinogen (Abtin et al., 2014; Pasparakis et al., 2014). In addition, vasodilation slows blood flow and causes congestion. As a result of the filling of red blood cells by the congestion, leukocytes are leaked. Especially, neutrophils infiltrate the site of infection to eliminate bacteria (Abtin et al., 2014; Pasparakis et al., 2014). In our experiment, histopathological analysis of SC tissue was performed to clarify the details of increased vascular permeability and the pathology dynamics during the infection. The congestion and red blood cells were not found immediately after infection at 0 h post-infection (Figure 3A), but the blood vessels were filled with red blood cells and became congested as the infection progresses (Figure 3A). Finally, red blood cells and leukocytes were decreased at the site of infection of end-stage mice at 12 h post-infection (Figure 3A). During the progression of this congestion, leukocytes appeared around the blood vessels at 3 h post-infection and peaked at 6 h, and then these were widely dispersed (Figure 3A). The leukocytes were positive for anti-neutrophil elastase antibody by the immunostaining from 3 to 12 h post-infection (Figure 3B). In comparison, no leukocytes were positive at 0 h post-infection (Figure 3B). Neutrophils, of which several parts were activated by the infection and labeled with anti-elastase, showed up at 3 h post-infection. They were increased, peaked at 6 h post-infection, and decreased after that in the SC tissue (Figure 3B). Thus, elastase release by infiltrated neutrophils at the infection site was shown to begin in the initial stage of infection. These aspects are known as cellulitis in *V. vulnificus* infection.

### Rapid Progression of Muscle Necrosis From Superficial to Deep Muscle

**Figure 4:**
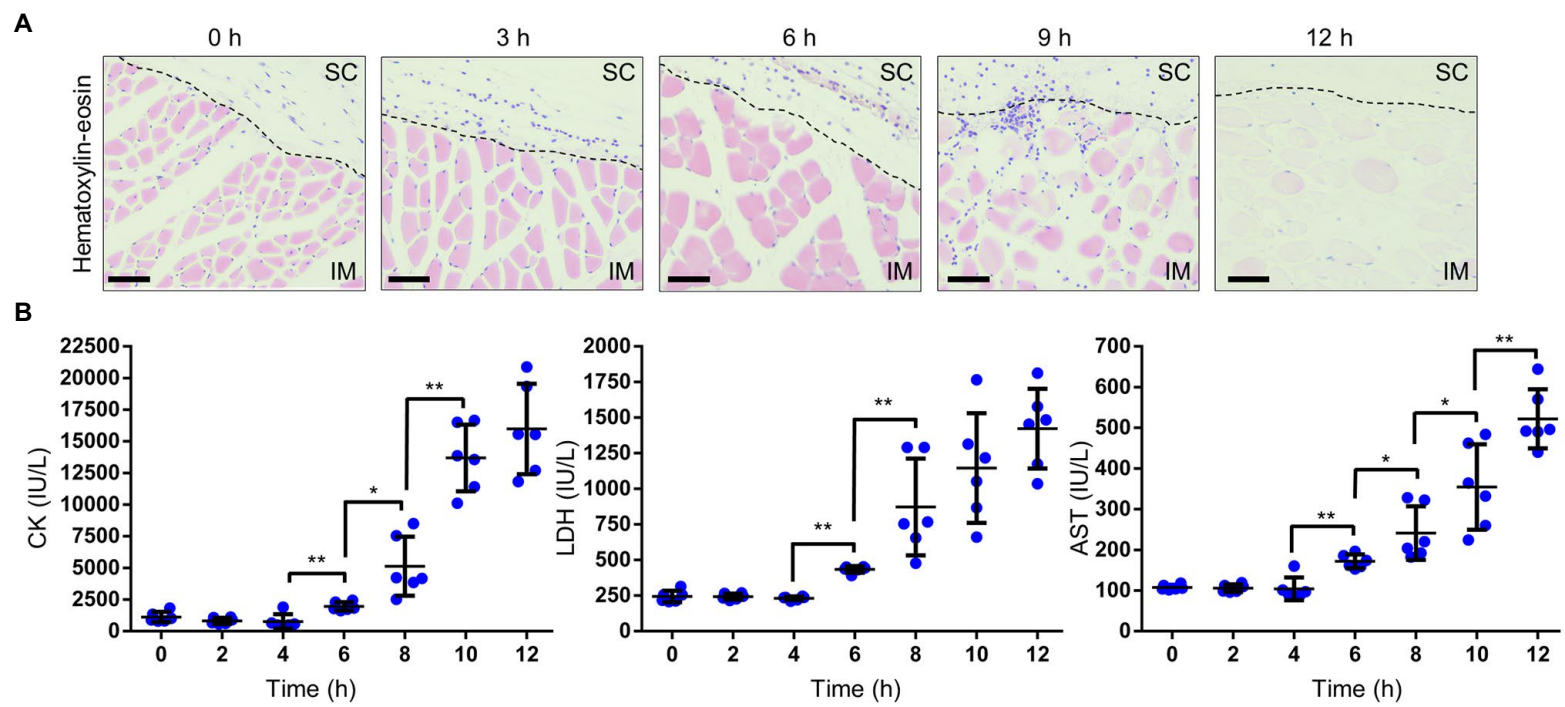
Necrosis in muscle. **(A)** Hematoxylin–eosin-stained histopathology images of muscle beneath the infection sites in a mouse infected with *Vibrio vulnificus* (10^6^ CFU/mouse). Scale bars: 25 μm. SC, subcutaneous; IM, intramuscular; and the black dotted line is the border between SC and IM. **(B)** Measurement of creatine kinase (CK), lactate dehydrogenase (LDH), and aspartate aminotransferase (AST) levels in the sera of mice infected with *V. vulnificus* (10^6^ CFU/mouse). Each symbol represents an individual mouse (*n* = 6/group). Error bars indicate SEM. ^*^p < 0.05, ^**^p < 0.01; Mann–Whitney *U*-test.

In NSTIs, necrosis is an irreversible pathological condition, occurred in fascia and muscle, and it progressed dramatically. In this study, the increased vascular permeability occurred along with the infiltration of neutrophils when blood vessels became congested (Figures 2A, 3B). Since the increased vascular permeability increased in the SC tissue, peaking in the muscle tissue, it was predicted that the increase resulted in pathological changes to muscle tissue. To investigate these, we performed a histopathological analysis. We observed the destruction of the fascial plane at the boundary between the SC tissue and the muscle, and muscle necrosis as reflected by enucleation and reduced dyeability with eosin at 6 h post-infection (Figure 4A). Moreover, these necrotic lesions (changes) of muscle tissue expanded from the surface to the deep muscle after that at 9–12 h post-infection (Figure 4A). Infiltration of neutrophils was also observed in muscle tissue at 9 h post-infection (Figure 4A).

We evaluated the severity of muscle necrosis by a biochemical test measuring levels of CK, LDH, and AST in the serum. These markers are released into the circulation when muscles are damaged. Levels of all markers began to increase at 6 h post-infection (Figure 4B). These results were consistent with the onset of muscle necrosis observed by histopathological analysis. After that, the levels increased in a time-dependent manner. These data, obtained from the murine wound infection model, could be used to help explain the fulminant progression of *V. vulnificus* NSTIs in patients.

### Bacterial Dynamics and Clinical Signs at the Primary Infection Site

**Figure 5:**
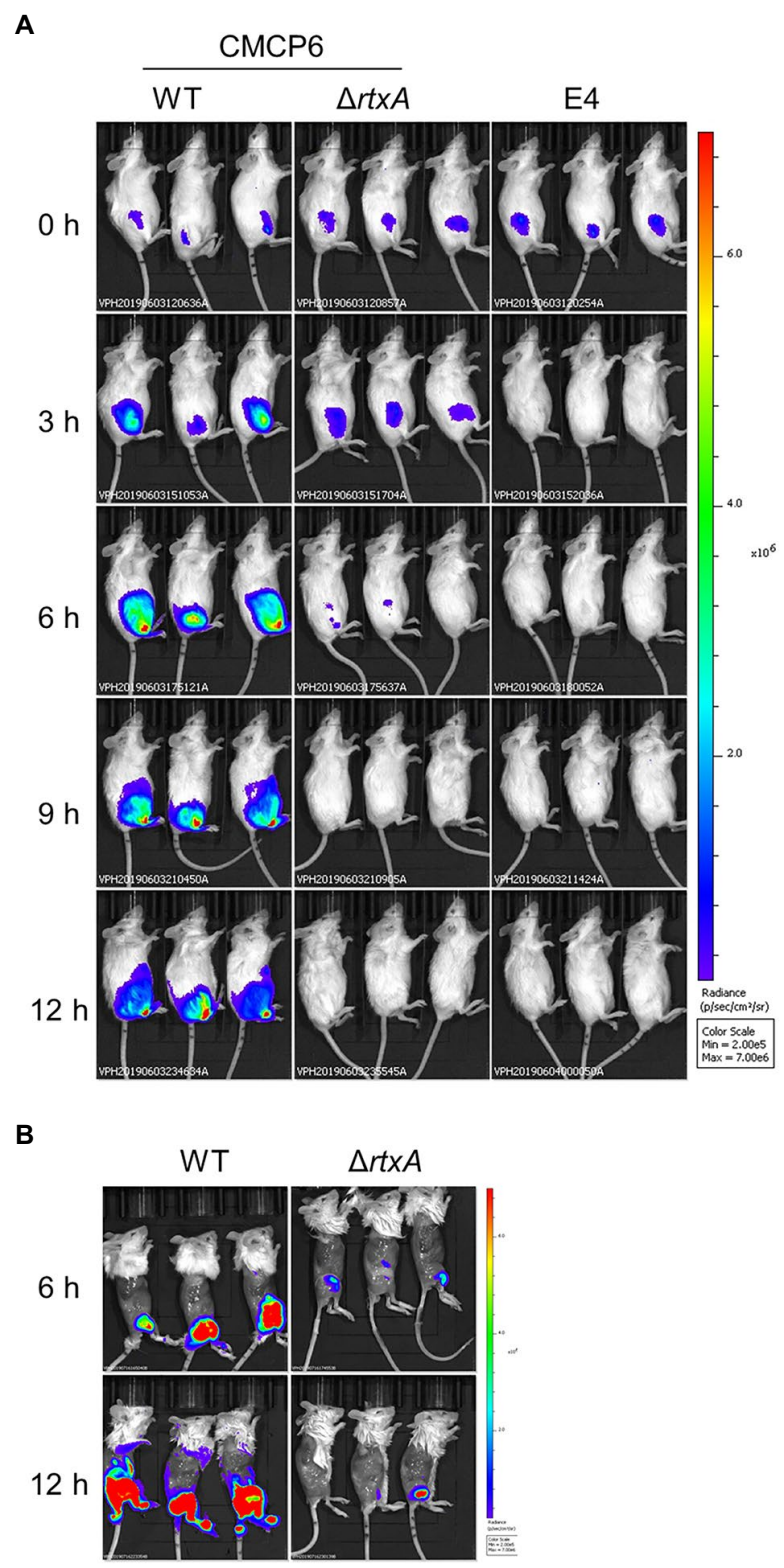
Spread and proliferation in soft tissues. **(A)** The luminescence signal of CMCP6 wild type (WT), CMCP6 Δ*rtxA*, and E4 detected by *in vivo* imaging system (IVIS) during 3 h time course (*n* = 6/group). **(B)** The luminescence signal of CMCP6 WT and Δ*rtxA* in the skinned mice detected by IVIS at 6 and 12 h post-infection (*n* = 6/group).

Our multilayer approach showed that clinical signs, such as cellulitis and necrotic fasciitis, emerge in the SC tissue in the early and evolve in deeper muscle tissue in the late stages of infection. We assess the proliferation associated with these clinical signs of CMCP6 WT and avirulent strains. To assess infection dynamics at the primary infection site, we infected mice with CMCP6 WT, Δ*rtxA*, and E4 expressing a luciferase operon and observed by IVIS (Figure 5A). The luminescence signals of CMCP6 WT were the most extensive at 6 h post-infection and were concentrated in the soft tissue of the thigh. On the other hand, CMCP6 Δ*rtxA* signals disappeared from 6 to 9 h post-infection, and E4 signals disappeared from 3 to 6 h post-infection from the soft tissue of the thigh (Figure 2A). These results indicate that CMCP6 WT colonizes and proliferates in the primary infection site, but CMCP6 Δ*rtxA* and E4 could not. Next, since the necrosis emerged in deeper muscle tissue in the late stages of infection, we skinned the mice to assess bacterial invasion into the muscle and observed by IVIS (Figure 5B). The luminescence signals of CMCP6 WT were extensive at 12 h post-infection in the muscle (Figure 5B). On the other hand, CMCP6 Δ*rtxA* signals completely disappeared at 9 h post-infection in the soft tissue, but some were detected from the muscle at 12 h post-infection (Figure 5B). We found that it was the CMCP6 WT strain infection in the skin and SC tissue that had spread, causing cellulitis and necrotizing fasciitis. Later, the WT strain invaded and proliferated in the muscle causing muscle necrosis. Almost all Δ*rtxA* strains were less virulent and infections were cleared from the skin and SC layers, suggesting that RtxA is essential for immune evasion and proliferation in the SC tissue.

### Bacterial Proliferation in the Muscle Lead to an Elevation of the Number of Bacteria in the Circulation

**Figure 6:**
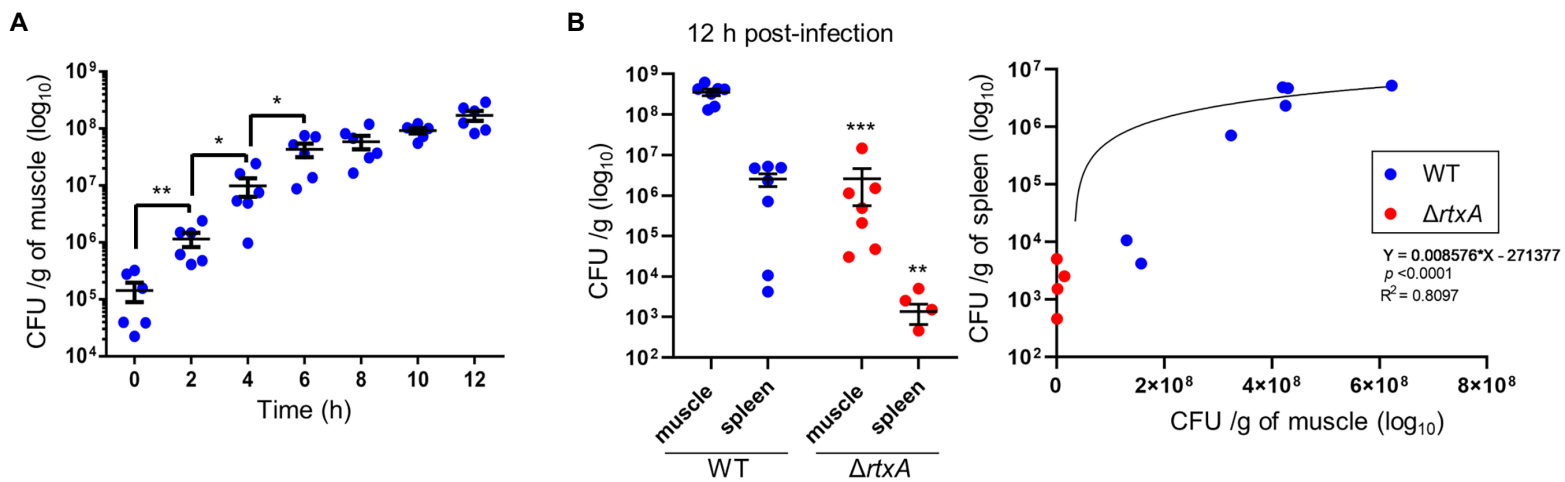
**(A)** Bacterial burdens in the muscle of mice subcutaneously inoculated with CMCP6 wild type (WT) calculated as CFU/g during 2 h time course. **(B)** Bacterial burdens in the muscle and the spleen of mice subcutaneously inoculated with CMCP6 WT, and Δ*rtxA* calculated as CFU/g at 12 h post-infection. Each symbol represents an individual mouse (*n* = 6/group). Error bars indicate SEM. ^*^p < 0.05, ^**^p < 0.01, and ^***^p < 0.001 compared with WT; Mann–Whitney *U*-test. Simple Linear Regression Analysis in the right panel of Figure 2C.

*In vivo* imaging system analysis showed that *V. vulnificus* invades and proliferate the muscle tissue. We calculated the bacterial burden of the muscle beneath the infection site. CMCP6 WT were detected immediately after infection, exponentially increased until 6 h post-infection, and continued to increase at 12 h post-infection in the primary infection site (Figure 6A). Since the progression of clinical signs and proliferation of *V. vulnificus* in the muscle tissue were suggesting the fatal condition of the host, we investigate the correlation of bacterial proliferation between the local infection site and systemic circulation, an index of sepsis of the host, and lethality of *V. vulnificus*, we inoculated the 10^6^ CFU/head of CMCP6 WT or Δ*rtxA* and calculated the bacterial burden of the muscle and the spleen. The number of bacteria collected from the muscle of CMCP6 WT-infected mice was finally 4.20 × 10^8^ CFU/g (median) at 12 h post-infection when the mice began to die (Figures 1, 2C). The number of bacteria collected from the spleen of CMCP6 WT-infected mice was 2.33 × 10^6^ CFU/g (median; Figure 6B). At the same time point, the number of bacteria collected from the muscle of mice infected with CMCP6 Δ*rtxA* was 4.87 × 10^5^ CFU/g (median) and significantly lower than that of CMCP6 WT (Figure 6B). Mainly, CMCP6 Δ*rtxA* was frequently not detected from the spleen in the infected mice (*n* = 3 of 8), and the number of bacteria collected from the spleen was 4.59 × 10^2^ CFU/g (median; Figure 6B). The correlation coefficient between the number of bacteria in muscle and the number of bacteria in the spleen was 0.8097 (Figure 6B). This strong positive correlation proved that the proliferation in the muscle was essential for increasing the number of bacteria in the systemic circulation, which is known as a representative aspect of septicemia in *V. vulnificus* infection, ultimately leading to host death.

### Muscle Invasion Critical for Lethality

**Figure 7:**
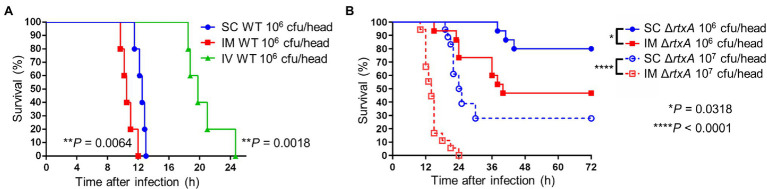
Critical invasion leading fatal outcome. **(A)** Kaplan–Meier survival curves for mice (*n* = 6/group) inoculated subcutaneously (SC), intramuscularly (IM), or intravenously (IV) with CMCP6 wild type (WT) and monitored for 72 h. ^**^p < 0.01 compared with SC inoculation; log-rank test. **(B)** Kaplan–Meier survival curves for mice (*n* = 12/group) inoculated SC or IM with CMCP6 Δ*rtxA* (10^6^ CFU/mouse and 10^7^ CFU/mouse) and monitored for 72 h. ^****^p < 0.0001, ^*^p < 0.05 compared with each dose of SC inoculation; log-rank test.

We compared survival times of mice receiving SC, intramuscular (IM), and IV injections. IM-inoculated mice survived for the shortest amount of time, followed by SC- and IV-inoculated mice (Figure 7A). Similarly, we compared the survival times of mice receiving SC and IM injections of Δ*rtxA*. Mice IM-inoculated with 10^6^ CFU/head of Δ*rtxA* showed higher mortality than SC-inoculated mice (Figure 7B). The high dose of Δ*rtxA* (infection with 10^7^ CFU/head) seemed to have been the cause of death in mice *via* the SC routes of infection (Figure 7B), although the survival time was delayed compared with mice infected with WT (10^6^ CFU/head; Figures 7A,B). However, mice IM-inoculated with 10^7^ CFU/head of Δ*rtxA* died as early as did mice infected with WT (Figures 7A,B). Thus, allowing the invasion into the muscle by *V. vulnificus*, even attenuated strains, is at a potent risk for the fatal outcome. The high bacterial burden in muscle led to a fatal outcome caused by *V. vulnificus* infection.

## Discussion

Adequate infection models that mimic human disease are the key to analyzing the mechanisms of bacterial pathogenesis. In the *V. vulnificus* wound infection; there are reports of cases of infection in healthy hosts (Dechet et al., 2008; Newton et al., 2012; Oliver, 2015). The first line of defense by the skin’s barrier function is destructed by wounds, allowing the bacteria to infect the hosts (Pasparakis et al., 2014). The short incubation period of the infection means that the *V. vulnificus* has a mechanism to colonize, proliferate, and spread by evading innate immunity in the intradermal and SC tissues of healthy hosts. This study elucidated the relationship between bacterial proliferation and pathophysiological progression in the murine wound infection model at each infection step.

Our wound infection model showed that a clinical isolate strain caused the lethal infection, whereas a strain lacking MARTX and an environmental strain lacking capsule were avirulent in the same dose (Figure 1). The MARTX is a major virulence factor of *V. vulnificus* (Jeong et al., 2000; Lo et al., 2011). Lo et al. (2011) showed that Δ*rtxA* was reduced in resistance to neutrophils in a model of skin air sac infection (Jeong et al., 2000). The MARTX, required for proliferation at the site of infection, enabled the killing of phagocytic cells, thus increasing cytotoxicity (Lo et al., 2011). The capsule is essential for resistance to phagocytic cells in various bacteria (Simpson et al., 1987; Oliver, 2015; Yamazaki et al., 2019; Kashimoto et al., 2021). The environmental strain E4 and Δ*rtxA* were a low ability to proliferate in the subcutaneous tissues compared with WT (Figure 5A). These results show that the innate immunity, including neutrophils in the wound infection model, could eliminate these attenuated strains of *V. vulnificus* in the soft tissues. Thus, our infection model enables the investigating of the mechanisms for evading the host immunity and proliferation during the wound infection.

The perivascular inflammation and excessive activation of neutrophils result in the release of neutrophil elastase, which also leads to increased vascular permeability (Park et al., 1996; Jeong et al., 2000; Johansson et al., 2009; Pasparakis et al., 2014; Siemens et al., 2016). Our wound infection model showed perivascular inflammation, infiltration of neutrophils, neutrophil elastase release, and increased vascular permeability during the infection (Figures 2–4). However, the significance of increased vascular permeability during the NSTIs has not been shown to date. Increased vascular permeability occurs in infection and lymphedema in the lower limbs, occurring due to clogging of lymph nodes with an increased risk of severe infection and onset of cellulitis (Baker, 1982; Simon and Cody, 1992; Swartz, 2004; Sarani et al., 2009; Gulzar et al., 2021). One cause of the high risk of infection in increased vascular permeability is that amino acids and proteins are leaked out of the blood vessel and served as a nutrient source for the proliferation of bacteria. Besides, exudate fluid accumulates in the tissues, facilitates swelling leading to the expansion of the tissue space. We have shown that the spread of *V. vulnificus* depends on flagellar-based motility and chemotaxis in the soft tissues (Yamazaki et al., 2020). The spread of *V. vulnificus* WT in soft tissues shown by IVIS was the most extensive at 6 h post-infection after maximizing increased vascular permeability in the subcutaneous tissue at 4 h post-infection (Figures 2A, 5A). Sequentially, the fluorescence signals in IVIS analysis gradually decreased from 6 to 12 h post-infection when vascular permeability of the subcutaneous tissue began to decrease (Figures 2A, 5A). In addition, the bacterial number of CMCP6 WT in muscle was increased following increased vascular permeability in muscle (Figures 2B, 6A; Table 1). These data suggested that *V. vulnificus* proliferates at the site where the vascular permeability is increased. This proliferation may be provided based on the sensing of nutrient sources and spread through the space induced by the increased vascular permeability. The increased vascular permeability caused by *V. vulnificus* infection plays a vital role in exacerbating the pathological condition of NSTI.

**Table 1:** Summary of *p* values from Mann–Whitney *U* test.

Figure	Measurement item	0 h	2 h	4 h	6 h	8 h	10 h	12 h
2A	Increased vascular permeability in the SC tissue	–	**0.0022**	**0.0043**	**0.0173**	0.0649	**0.0043**	0.2403
2B	Increased vascular permeability in the muscle tissue	–	**0.0411**	**0.0022**	**0.0043**	0.4848	**0.0043**	**0.0087**
4B	CK	–	0.3095	0.1797	**0.0260**	**0.0022**	**0.0022**	0.3939
4B	LDH	–	0.6667	0.5628	**0.0022**	**0.0022**	0.2229	0.2403
4B	AST	–	0.6169	0.2403	**0.0087**	**0.0152**	**0.0260**	**0.0087**
6A	Bacterial burden in the muscle tissue	–	**0.0022**	**0.0152**	**0.0260**	0.4848	0.1320	0.1320

*Bold values show a significant difference. The *p* values are the results of a comparison between the value at that time with the value at the previous time*.

Necrosis is a characteristic pathological condition in *V. vulnificus* NSTI but has never been reproduced in mouse models. In this study, we successfully reproduced the progression of necrosis from superficial layers to deep muscle tissue (Figure 4A). This progressive necrosis could be confirmed by a biochemical blood test (Figure 4B). Our wound infection model can faithfully mimic the pathological conditions observed in humans. In summary, the murine wound infection model will help elucidate distinct mechanisms and virulence factors that allow *V. vulnificus* to evade host immunity, proliferate, and cause the pathologies.

In the treatment for NSTI, extensive surgical debridement of the local infection site exhibiting multiple lesions and necrosis is generally performed to reduce the number of bacteria and prolong a patient’s life (Swartz, 2004; Sarani et al., 2009; Stevens and Bryant, 2017). However, until our present study, no reports demonstrated that the proliferation of bacteria in the infection site and the systemic circulation correlate strongly and lead to host death. We showed the proliferation of *V. vulnificus* in soft tissue by IVIS and measurement of bacterial burden (Figures 5A, 6). In addition, CMCP6 WT invaded in muscle and spleen (Figure 6). In contrast, CMCP6 Δ*rtxA* was detected from the primary infection site by measurement of bacterial burden but could not be detected 9 h after infection by IVIS (Figures 5A, 6). Consistently, all mice infected with the WT strain died 12 h after infection, whereas some mice infected with Δ*rtxA* survived (Figure 1). In our infection model, the host died when bacterial proliferation in muscle was over 10^8^ CFU/g and around 10^4^ CFU/g in the systemic circulation (spleen; Figure 6). These indicate that the insufficient proliferation of CMCP6 Δ*rtxA* was not enough to increase the number of bacteria in the spleen. However, Δ*rtxA* has the potential to invade and proliferate in the muscle tissue depending on other mechanisms associated with the lethality. This point out was supported by the death of mice by the high dose of inoculation or by IM-inoculation of Δ*rtxA* (Figure 7A). Our data ensure the significance and effectiveness of the debridement of muscles at the primary infection site, especially when the infection is due to a deep wound, to prevent progression to lethal septicemia. Investigating the mechanisms for immune evasion and efficient proliferation in the muscle tissue is required to elucidate the pathogenic mechanisms for the process leading to sepsis.

## Data Availability Statement

The original contributions presented in the study are included in the article/supplementary material; further inquiries can be directed to the corresponding author.

## Ethics Statement

The animal study was reviewed and approved by Kyoko Shimabukuro The president of Kitasato University.

## Author Contributions

KYa and TKad conducted experiments. KYa, TKas, and KYo designed the study. KYa analyzed data. KYa and TKas wrote the manuscript. SU supervised the study. All authors contributed to the article and approved the submitted version.

## Funding

This work was supported by the Japan Society for the Promotion of Science KAKENHI under Grant number 18H02350. The foundation had no role in study design, data collection, and interpretation, or the decision to submit the work for publication. The authors have no additional financial interests.

## Conflict of Interest

The authors declare that the research was conducted in the absence of any commercial or financial relationships that could be construed as a potential conflict of interest.

## Publisher’s Note

All claims expressed in this article are solely those of the authors and do not necessarily represent those of their affiliated organizations, or those of the publisher, the editors and the reviewers. Any product that may be evaluated in this article, or claim that may be made by its manufacturer, is not guaranteed or endorsed by the publisher.
